# Sudden Fall in the Lipid-Lowering Effect of Evolocumab: The Butler Is Not Always Guilty

**DOI:** 10.3390/medicina57090857

**Published:** 2021-08-24

**Authors:** Federica Fogacci, Claudio Borghi, Antonio Di Micoli, Arrigo F. G. Cicero

**Affiliations:** 1 Hypertension and Cardiovascular Risk Factors Research Group, Medical and Surgical Sciences Department, University of Bologna, 40138 Bologna, Italy; federica.fogacci@studio.unibo.it (F.F.); claudio.borghi@unibo.it (C.B.); 2 Department of Heart, Lung and Vessels, IRCCS Azienda Ospedaliero-Universitaria di Bologna, 40138 Bologna, Italy; antonio.dimicoli@aosp.bo.it

**Keywords:** PCSK9 inhibitor, evolocumab, drug therapy problems

## Abstract

A 78-year-old man came to our attention after undergoing coronary computed tomography angiography documenting multivessel coronary artery disease. He was started on treatment with the proprotein convertase subtilisin/kexin type 9 (PCSK9) inhibitor evolocumab 140 mg subcutaneously every 2 weeks. Treatment-emergent changes in lipids and lipoproteins were long-lasting, and the medication was well tolerated by the patient in the long-term. Unexpectedly, after 2 years of continuous treatment with evolocumab, serum lipids increased, apparently without any reasonable explanation. During the follow-up visit, the patient was found to have habitually injected evolocumab into his right thumb instead of into the appropriate injection sites (i.e., abdomen, thighs or upper arms) after turning the injector upside down.

## 1. Introduction

The incidence of drug therapy problems (DTPs) has significantly increased over the past few decades [1]. Worldwide, more than half of all medications are prescribed and dispensed inappropriately, and half of the patients fail to take them correctly [2,3]. This represent a relevant issue, especially with regards to all the chronic diseases that frequently recur when patients are off-therapy or not adequately managed with the standards of care.

DTPs can compromise the quality of life, increase the risk of hospitalization and overall health care costs [4]. Moreover, DTPs not infrequently lead to death when they are not detected early and quickly resolved [4].

Below, we describe the case of a patient who experienced an absolutely unexpected increase in serum lipids after 2 years of continuous treatment with the proprotein convertase subtilisin/kexin type 9 (PCSK9) inhibitor evolocumab.

## 2. Case Presentation

A 78-year-old non-smoking Caucasian man came to our attention for the first time after undergoing coronary computed tomography angiography documenting multivessel coronary artery disease. He had consistently high levels of low-density lipoprotein cholesterol (LDL-C) and lipoprotein(a) (Lp(a)) and a family history of early-onset CVD in first-degree relatives. Comorbidities were chronic myeloid leukemia (CML), recurrent prostate cancer, hypertension with left ventricular hypertrophy (LVH), recurrent deep-vein thrombosis (DVT), non-alcoholic fatty liver disease (NAFLD; FibroScan fibrosis score F0 to F1) and Type 2 diabetes. For these reasons, by the time the patient was on pharmacological treatment with 500 mg metformin twice a day; triptorelin, 75 mg acetylsalicylic acid, 40 mg pantoprazole, cholecalciferol, 5 mg amlodipine, 16 mg candesartan, 2.5 mg indapamide and 2.5 mg bisoprolol twice a day; and ezetimibe. He was treated with an intermittent imatinib schedule (alternate months on and off imatinib). He had a personal history of intolerance to statins even at the lowest doses because of disabling muscle cramps and increased creatine kinase (CK) serum levels judged to be clinically relevant. These symptoms disappeared after withdrawal of statins and recurred after rechallenge with statins, supporting a causal effect.

He was started on treatment with the PCSK9 inhibitor evolocumab, 140 mg subcutaneously (SC) every 2 weeks (QW2). Treatment-emergent changes in lipids and lipoproteins were long-lasting (Table 1) and consistent with our previous observations [5], and the medication was well-tolerated by the patient in the long term and correctly assumed over time. Unexpectedly, after 2 years of continuous treatment with evolocumab, serum lipids increased (Table 1), apparently without any reasonable explanation.

The possibility that the patient may have developed an acute condition potentially reducing the effect of evolocumab had to be investigated. The most plausible explanation for the increase in serum lipids was that the patient had thyroiditis. We planned additional laboratory tests, but his hormonal thyroid test was normal (thyroid stimulating hormone = 2.30 μU/mL) and thyroid antibodies were negative.

We then speculated that the patient missed the drug injection, even though he was firmly claiming that he had regularly taken the drug. After discussing with the hospital pharmacy service director who was responsible for the drug utilization review (DUR), we were able to ascertain that the patient had not received fewer injections than prescribed. Moreover, the patient’s wife confirmed that her husband was very careful to administer the drug in the right time window (i.e., once every 14 days). However, even though the patient was supposed to have been administered evolocumab the day before the visit, on physical examination, he did not have any puncture marks into the appropriate injection sites (i.e., abdomen, thighs or upper arms), suggesting no recent administration of the drug in those areas. On the contrary, an injection mark was clearly distinguishable on the patient’s right thumb.

**Figure 1:**
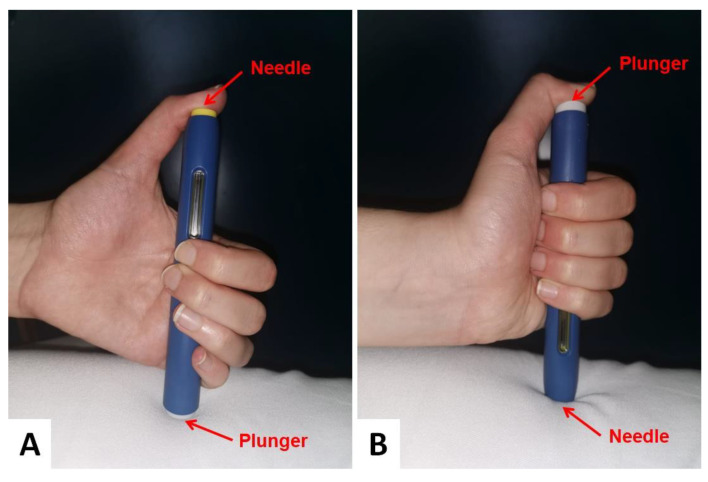
(**A**) Correct and (**B**) incorrect method of evolocumab administration.

By repeating the drug administration procedure with a sham injection during the follow-up visit, the patient was found to have habitually injected evolocumab into his right thumb after turning the injector upside down (Figure 1).

After retraining the patient on proper care, the levels of plasma lipids fell back again (total cholesterol = 106 mg/dL; LDL-C = 25 mg/dL; Lp(a) = 49.7 mg/dL) and our hypothesis was definitely confirmed.

## 3. Discussion

The active participation of the patients in the care process is important to enhance their clinical management and minimize the risk of complications linked to pharmacological treatment [6].

The OSLER-1 trial (Open-Label Study of Long-Term Evaluation Against LDL-C Trial) has already showed the consistent LDL-C–lowering effect of evolocumab in the long-term, with no neutralizing antibodies detectable [7]. For this reason, there was apparently no reasonable explanation justifying the observation in our patient. However, a closer analysis allowed us to identify a DTP that made the PCSK9 inhibitor less effective than expected [8].

Our case report highlights the importance of identifying all patient-level variables that are potentially able to affect the efficacy of even the most powerful lipid-lowering drugs, such as evolocumab.

Similar to what has already been demonstrated for the treatment of hypertension, in lipidology, the failure to account for all the dimensions of adherence also typically leads to suboptimal improvements in the efficacy parameters and in the associated clinical outcomes [9]. As regards our case study, the rapid identification of the underlying reason for the increase in serum lipids levels spared the patient from undergoing further expensive and not strictly useful laboratory tests and/or instrumental examinations to identify the clinical reason for the reduction in the effectiveness of evolocumab.

Certainly, the identification and documentation of DTPs and the predictors are crucial to design preventive strategies.

A recent analysis found that the risk of DTPs increases with the use of more than three drugs in patients affected by CVD [10]. Actually, our patient took a number of different medications for multiple chronic conditions, and this is likely to have contributed to the final observation.

Another factor to be considered is the need for periodic retraining in the case of long-lasting treatments, especially for elderly and frail patients. This is particularly relevant when prescribing a high-cost drug not requiring serial therapeutic monitoring, since the effect of the drug on the laboratory parameters is not repeatedly evaluated at close range. In patients under stabilized treatment with evolocumab, for example, only one laboratory assessment every 6 months is required for prescription renewal in Italy. In this case, if our patient had poorly administrated the drug for 6 months, his serum lipid levels would have further increased over time, and the positive effects of the drug would be definitely reduced.

## Figures and Tables

**Table 1:** Serum lipids concentration of the patient.

Parameters	Before Starting Treatment with Evolocumab	During Treatment with Evolocumab (Correct Administration of the Drug)		After Incorrect Administration of Evolocumab		
	Absolute Value (mg/dL)	Absolute Value (mg/dL)	Change Compared with Baseline (%)	Absolute Value (mg/dL)	Change Compared with Baseline (%)	Difference with Respect to Correct Administration (%)
Total Cholesterol	183	114	−37.7	122	−33.3	+4.4
Triglycerides	101	128	+26.7	102	+1	−25.7
HDL Cholesterol	60	62	+3.3	62	+3.3	0
Non-HDL Cholesterol	123	52	−57.7	60	−51.2	+6.5
LDL Cholesterol	102.8	26.4	−74.3	39.6	−61.5	+12.8
Lipoprotein(a)	59.1	59.8	+1.2	73.7	+24.7	+23.5

HDL = high-density lipoprotein; LDL = low-density lipoprotein.

## Data Availability

Data are contained within the article.
